# Abundance of Multidrug Resistance Efflux Pumps in the Urinary Metagenome of Kidney Transplant Patients

**DOI:** 10.1155/2020/5421269

**Published:** 2020-03-12

**Authors:** Asha Rani, Ravi Ranjan, Ahmed A. Metwally, Daniel C. Brennan, Patricia W. Finn, David L. Perkins

**Affiliations:** ^1^Department of Medicine, University of Illinois at Chicago, Chicago, IL 60612, USA; ^2^Department of Bioengineering, University of Illinois at Chicago, Chicago, IL 60612, USA; ^3^Department of Medicine, Johns Hopkins School of Medicine, Baltimore, MD 21287, USA; ^4^Division of Pulmonary, Critical Care, Sleep, and Allergy, Department of Medicine, University of Illinois at Chicago, Chicago, IL 60612, USA; ^5^Department of Microbiology and Immunology, University of Illinois at Chicago, Chicago, IL 60612, USA; ^6^Division of Nephrology, Department of Medicine, University of Illinois at Chicago, Chicago, IL 60612, USA; ^7^Department of Surgery, University of Illinois at Chicago, Chicago, IL 60612, USA

## Abstract

Antibiotic resistance including the emergence of multidrug resistant microbes has become a public health crisis. In this study, we analyzed the antibiotic resistance genes (ARGs) in the urinary metagenome of the kidney transplant and healthy subjects using metagenomic shotgun sequencing. Our data suggest an increased abundance of antibiotic resistance genes in the kidney transplant subjects. In addition, the antibiotic resistance genes identified in the transplant subjects were predominantly composed of multidrug efflux pumps (MDEPs) which are evolutionarily ancient, commonly encoded on chromosomes rather than plasmids, and have a low rate of mutation. Since the MDEPs had a low abundance in the healthy subjects, we speculate that the MDEPs may enhance the fitness of bacteria to survive in the high stress environment of transplantation that includes multiple stressors including surgery, antibiotics, and immunosuppressive agents.

## 1. Introduction

Antibiotic resistance has emerged as a major medical and public health challenge which has been driven, in part, by the misuse of antibiotics in both the clinical and agricultural arenas [1, 2]. Exposure to clinical levels of antibiotics can promote mutations inducing enhanced antibiotic resistance and the emergence of multidrug resistant strains. Following kidney transplantation, infections remain a major complication, and the most common type of acute infection following solid organ transplantation is bacterial [3, 4]. Consequently, subjects are frequently treated with prophylactic antibiotics, for example, sulfamethoxazole-trimethoprim, which we previously reported can induce increased levels of antibiotic resistance genes (ARGs) in the urinary metagenome [5]. Specifically, we reported increased levels of dihydrofolate synthase/folylpolyglutamate synthase, two enzymes that can increase folate production but are not blocked by sulfamethoxazole-trimethoprim in subjects that developed urinary tract infections following transplantation.

There are multiple mechanisms for the selection and development of ARGs, but the contribution of the various mechanisms has not been clearly elucidated. Based on the genetic analysis of bacterial genomes, it is estimated that there are more than 20,000 antibacterial resistance genes (ARGs) comprising approximately 400 different types [6]. ARGs are evolutionarily ancient and were present before the advent of antibiotics indicating that ARGs have biological functions in addition to inhibiting effects of antibiotics. In vitro studies have shown that ARGs can inhibit transcription and modulate metabolism suggesting important biological functions in natural ecosystems [7]. Thus, the contribution of antibiotics and resistance to antibiotics is not well understood in terms of resilience or susceptibility to adverse outcomes including infection or graft rejection in kidney transplantation.

One of the most studied types of ARGs is the beta-lactamases that produce resistance to penicillin. Beta-lactamases are usually expressed on plasmids and can generate new specificities of resistance within days due to the accumulation of point mutations. In contrast, another class of ARGs is the multidrug efflux pumps (MDEPs), which are encoded in the bacterial chromosomes, expressed in all members of a species, and mutate slowly, and thus are not selected by exposure to antibiotics. The MDEPs are ancient genes present in prokaryotes, archaebacteria, and eukaryotes including mammals. An important example in clinical medicine is p-glycoprotein, which can be responsible for resistance to chemotherapy agents. There are five types of MDEPs in bacteria which have diverse functions including quorum sensing and detoxification of heavy metals, bile salts, and bacterial metabolites. Since the MDEPs emerged early in evolution their original functions were not to convey resistance to antibiotics; nevertheless, the efflux pumps are effective mechanisms to generate antibiotic resistance [8, 9].

To investigate the spectrum of ARGs within the kidney transplant and healthy subjects, we employed shotgun metagenomic sequencing (MGS) in the urinary metagenome. In contrast to targeted16S rRNA amplicon sequencing, MGS sequencing identifies nearly complete genomes that can identify bacterial taxa at the species level and thus annotate the complete array of ARGs. Interestingly, our study identifies major changes in the profile of ARGs in kidney transplant subjects.

## 2. Material and Methods

### 2.1. Study Design, Ethics Statement, Metagenome DNA Isolation, and Sequencing

The objective of this study was to investigate the antibiotic resistance genes (ARGs) contributed by the urinary microbiome of kidney transplant recipients and healthy subjects using shotgun metagenomic sequencing. As previously described, urine samples were collected posttransplant from kidney transplant recipients and controls [5] and 17 additional controls described in [Supplementary-material supplementary-material-1]. Similar to the DNA isolation method, library preparations were performed and were sequenced on Illumina MiSeq with 301 paired end chemistry. This study was approved by the Washington University School of Medicine Institutional Review Board, St. Louis, Missouri (IRB ID # 201102312, Protocol Number # 07-0430) and by the University of Illinois Institutional Review Board, Chicago, Illinois (IRB # 2014-1227).

### 2.2. Bioinformatic Analyses and Antibiotic Resistance Gene Annotation

Raw reads from transplant and control samples were quality filtered and trimmed to remove human sequences using CLC genomics workbench default parameters (CLC bio, Qiagen). High quality filtered reads were *de novo* assembled into contigs, and reads were mapped back to contigs to generate the number of reads per contig. The sequence data was aligned with the antibiotic resistance genes in the ARDB (Antibiotic Resistance Genes Database) using 80% identity and *e*-value of 1*e*^−5^ over 80% alignment length of the ORF/gene [6]. ARDB allows the search against antibiotic resistance genes, type, organism, and antibiotic class. A csv file format with antibiotic gene, bacterial taxonomy, and antibiotic class associated with each sample was retrieved. The data file was normalized by dividing the number of reads aligned to each resistance gene/total reads used in the analysis and was converted to a square data matrix for further analysis. Data corresponding to both functional and taxonomical distributions were analyzed using MG-RAST SEED database using 80% similarity cutoff [10].

### 2.3. Statistical Analyses

Principal Coordinate Analysis (PCoA) was performed to find the axis that best explains the variance in the ARGs data set. The PCoA plot was generated using the Bray-Curtis Dissimilarity matrix for ARGs abundance between transplant and control group. In order to test for significant differences in the transplant and control group, a one-way analysis of similarity (ANOSIM) was conducted as described [11]. SIMPER (Similarity Percentage) analysis was performed to find the ARGs which contributed towards the % dissimilarity among the groups. ANOSIM and SIMPER analyses were performed using PAST statistical software v 3.0 [12]. Differences between two groups were assessed using the Mann–Whitney *U* Test and Wilcoxon Rank Test. A *p*-value of ≤0.05 considered as a significant difference after multiple test corrections with Benjamini–Hochberg false discovery rate (FDR). Data sets that involved more than two groups were assessed by analysis of variance (ANOVA) followed by Tukey's multiple comparison *post hoc* tests. Data were analyzed using GraphPad Prism (GraphPad Software, San Diego, CA, USA) and were considered as statistically significant with *p* ≤ 0.05 unless otherwise indicated.

### 2.4. High-Throughput Sequencing Data Availability

The sequence data for the study has been submitted to the MG-RAST web server under the following accession number provided in [Supplementary-material supplementary-material-1].

## 3. Results

**Figure 1:**
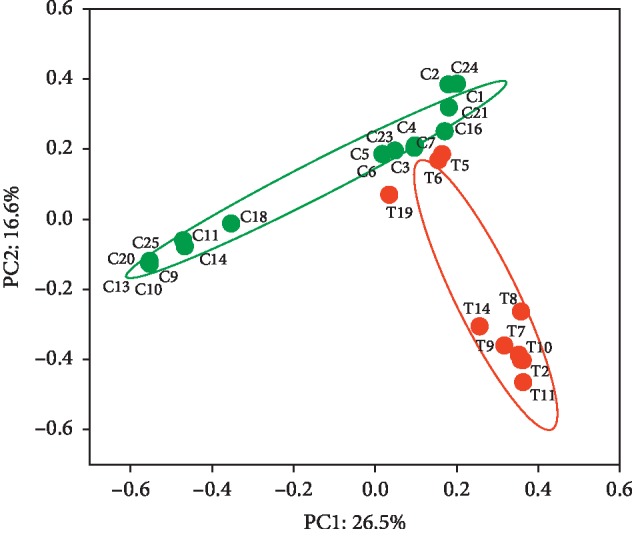
Principal Coordinate Analysis. Antibiotic resistance gene (ARG) based profile for each sample is generated based on the Bray-Curtis similarity matrix in kidney transplant and control groups. The first two components (PC1 and PC2) of the PCoA plot explained 26.5 and 16.6% variations, respectively, in transplant and the control group, with a wider range of within-group distribution. Similar direction and magnitude of clustering at 90% CI, indicate a positive association. The percentage of total variance explained by each axis is noted in both the axis labels. Red circles represent the transplant, and green circles represent control group samples. (C: Control, T: Transplant).

Transplant subjects are exposed to multiple stressors including surgery, immunosuppression, and antibiotics. We analyzed the abundance of antibiotic resistance genes (ARGs) in the urinary metagenome in kidney transplant recipients versus controls (Table 1). We performed metagenomic shotgun sequencing (MGS) of additional 17 controls for this study, which generated a total of 312 × 10^6^ raw reads. The raw reads were filtered to remove human sequences and were assembled into 1,429,333 contigs ([Supplementary-material supplementary-material-1]). We identified ARGs in 10 out of 21 transplants, and 19 out of 25 controls. To compare the metagenomes of the kidney transplant and control subjects, we performed Principal Coordinate Analysis (PCoA) which projected separation of the two groups based on the ARGs identified in each sample with 26.5% of the variance defined by the first principal coordinate (Figure 1). Variance of the control group ARGs profile was primarily encompassed in the first principal coordinate, whereas the variance of the transplant resistome was predominantly within the second principal coordinate.

**Figure 2:**
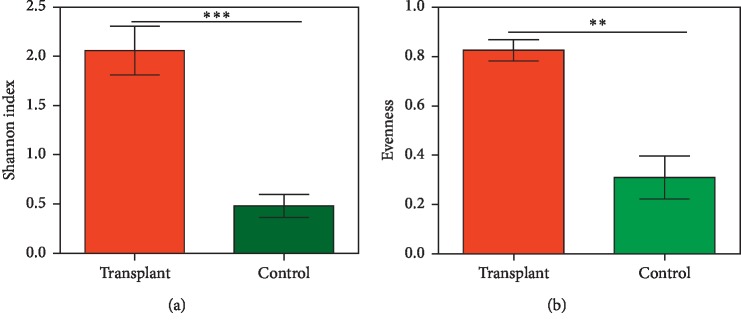
Diversity indices for ARG richness and evenness. Diversity indices were compared between the transplant (*n* = 21) and control (*n* = 25) groups using ARG data. (a) Shannon diversity index, (b) Evenness. The significant differences among the groups were computed using Mann–Whitney *U* Test (^*∗∗*^*p* ≤ 0.001, ^*∗∗∗*^*p* ≤ 0.0001).

We compared the relative abundance of the genera of the two groups with the *Wilcoxon* rank test which showed different ranks in the groups (*p* ≤ 0.05) (Table 1). Of note, the genus *Enterococcus*, which we previously reported to express genes predicting resistance to sulfamethoxazole-trimethoprim [5] comprised 28.6% of the transplant microbiome, but only 7.2% of the control microbiome. In contrast, the *Propionibacterium* genus comprised 10.9% of the control microbiome, but only 4.8% of the transplant microbiome. Overall, 4 genera were unique to the transplant microbiome and 4 genera were unique to the control microbiome, and 8 genera were detected in both microbiomes (Table 1). We also analyzed the diversity in the ARGs among the groups. The Shannon diversity index (based on ARG abundance) was significantly higher in the transplant group at 2.1 versus 0.4 in the control group (*p* ≤ 0.001); similarly, the evenness was greater in the transplant group (Figures 2(a) and 2(b), respectively).

**Figure 3:**
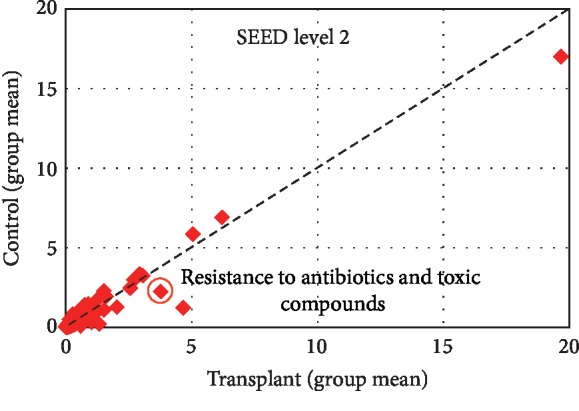
Functional analysis using SEED Subsystems at Level 2. Scatter plot was generated using the functions at level 2 among the groups. Pearson correlation between the control group (*y*-axis) and transplant group (*x*-axis).

**Figure 4:**
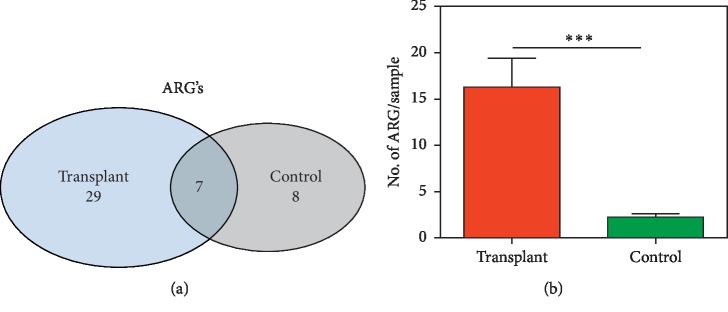
Detection of core antibiotic resistance genes (ARGs). (a) Venn diagram of unique and shared ARG between the transplant and control groups. (b) The number of ARG detected per sample is significantly higher in the transplant group compared to controls. Significant differences among the groups were computed using Mann–Whitney *U* Test (^*∗∗∗*^*p* ≤ 0.0001).

**Figure 5:**
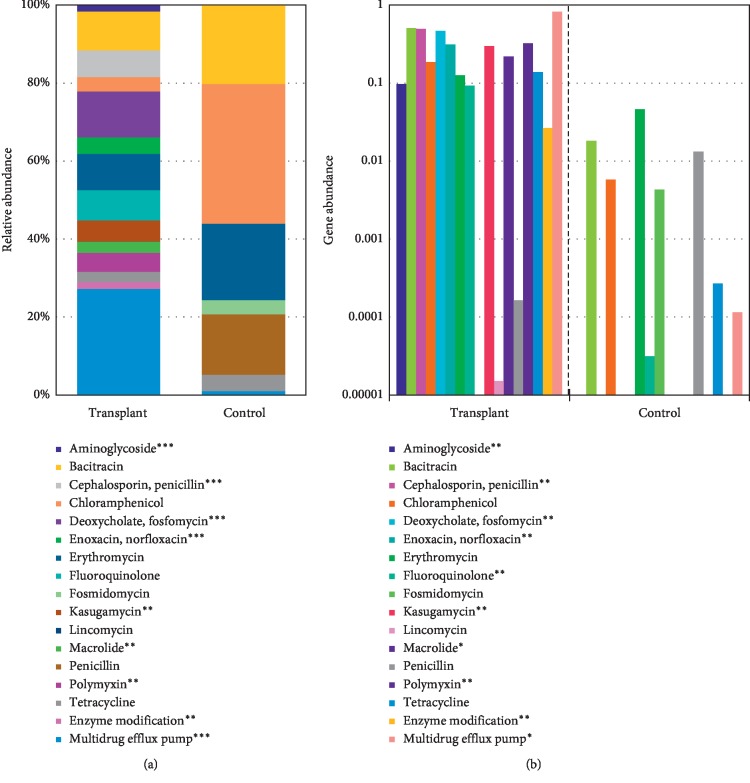
Abundance of ARG in transplant and control. (a) Relative abundance of antibiotic class in each group. (b) Absolute count of antibiotic class in each group. The significant differences between the groups were computed using Wilcoxon Rank Test (*p* ≤ 0.05).

To identify potential differential functions in the transplant and control metagenomes, we employed the SEED database in MG-RAST v2.0 to identify the relative abundance of ARGs. Analysis of level_1 did not detect significant differences; however, analysis of the more specific level_2 of MG-RAST detected increased “Resistance to antibiotics and toxic compounds” in the transplant group (Figure 3). Overall, we detected 29 unique ARGs in the transplant group, 8 unique in the control group, and 7 that were detected in both groups (Figure 4(a)). The mean number of unique ARGs per patient was 16 in the transplant group, but only 2 in the control group (Figure 4(b)). Next, we determined the predicted spectrum of antibiotic resistance genes based on the metagenomes in each group. Analysis of the relative abundance of ARGs indicated a more diverse profile in the transplant subjects compared with control subjects (Figure 5(a)). In addition, a comparison of the specificity of the resistance profiles showed different profiles in the two groups (Figure 5(b)). For example, the transplant subjects indicated resistance to 16 of the 17 antibiotics class analyzed. In contrast, the control subjects indicated resistance to a subset of the antibiotics that included bacitracin, chloramphenicol, erythromycin, fluoroquinolone, fosmidomycin, penicillin, and tetracycline. In addition to these antibiotics, the transplant subjects were also resistant to aminoglycosides, cephalosporins, fosfomycin, norfloxacin, kasugamycin, lincomycin, macrolides, and polymyxin. Thus, transplant subjects have a broader profile of antibiotic resistance and a greater abundance of antibiotic resistance genes that is often an order of magnitude greater than in the control subjects.

**Figure 6:**
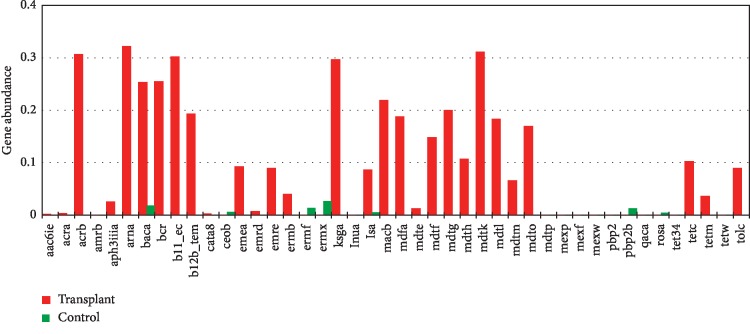
Bar chart representation of the ARG in transplant and control. Average abundance of ARG for transplant and control group. ARGs above 0.1% gene abundance are considered highly abundant.

**Figure 7:**
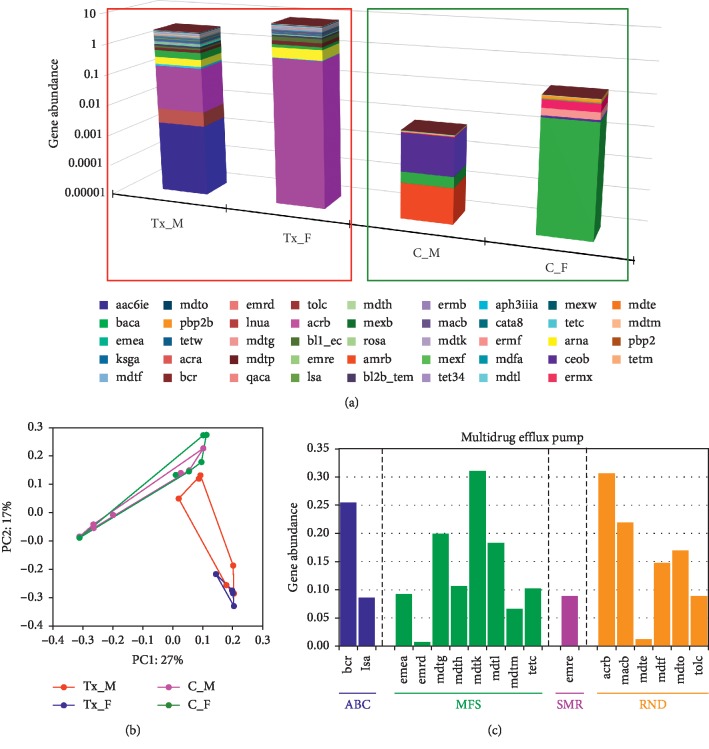
(a) Gender based ARG profile among transplant male (Tx_M) vs. females (Tx_F), and control male (C_M) vs. females (C_F). (b) Principal Coordinate Analysis using Bray–Curtis similarity matrix in kidney transplant and control groups. (c) Major types of multidrug efflux pumps (MDEP) identified in the transplant group.

Since we employed MGS sequencing we were able to identify specific genes in the microbiota. Although some ARGs were detected at low levels, we detected a total of 44 ARGs from both groups, 36 were detected in transplant, and 15 in the control group, both groups shared 7 ARGs (Figure 6). Only 3 ARGs were abundant in the control subjects: *ermf* (a methyl transferase), *pbp2b* (beta-lactamase), and *rosa* (efflux pump/potassium antiporter); however, all 3 were identified at extremely low absolute levels in the transplant group. Interestingly, 14 (above 0.1 gene abundance) unique ARGs were identified in the transplant urinary metagenome suggesting a diverse baseline repertoire of ARGs. Since gender can affect susceptibility to urinary tract infections and has been shown to modify the urinary microbiome [13], we also compared the effect of gender in the 2 groups (Figure 7(a)). We detected changes associated with gender in both the transplant and control groups; however, a Principal Coordinate Analysis showed that the strongest association was between transplant versus control (Figure 7(b)). The SIMPER analysis showed that mean rank (based on Bray-Curtis Dissimilarity matrix) was not significantly different among the transplant male and female (ANOSIM *R* = 0.02, *p*=0.38), or the control male and female (ANOSIM *R* = 0.04, *p*=0.66). However, the mean rank between the transplant and control groups was significantly different (ANOSIM *R* = 0.59, *p* ≤ 0.001) compared to within-group difference ([Supplementary-material supplementary-material-1]).

An analysis of the type of resistance mechanisms of the ARGs in the transplant subjects indicated that the most common type was a multidrug efflux pump. A SIMPER analysis identified the ARGs which contributed the most differences among the groups, only ARG which contributed above 1% difference are shown (Table 2). In fact, 18 of the 36 ARGs in the transplant group were multidrug efflux pumps (MDEP). The SIMPER analysis showed that MDEPs contributed 65.7% towards the dissimilarity among the groups (Table 2).

There are five major types of MDEPs including ABC (ATP binding cassette), MATE (toxic compound extrusion family), MFS (major facilitator super family), SMR (small multidrug resistance family), and RND (resistance/nodulation division family). Next, we determined which type of efflux pumps were expressed in the transplant subjects (Figure 7(c)). Interestingly, we identified MDEPs from 4 of the 5 types of efflux pumps including 2, 0, 8, 1, and 6 from the ABC, MATE, MFS, SMR, and RND types, respectively. Thus, the MDEPs were derived from multiple diverse types of efflux pumps.

## 4. Discussion

In this study, we analyzed antibiotic resistance genes in a cohort of kidney transplant subjects compared with controls using metagenome shotgun sequencing (MGS). In a previous report, we showed that bacterial phyla were markedly different in transplant and control subjects [5]. In this study analysis of the bacterial genera showed significantly different abundances of the taxa in the transplant and control groups. For example, the transplant group had increased *Enterococcus* but decreased *Propionibacterium*, *Neisseria*, and *Bacteroides*. In addition, 4 other genera were detected in the transplant group that were absent in the controls (Table 1). In the current study, our analysis of the ARGs by PCoA indicated distinct subsets of ARGs in the transplants and the controls (see Figure 1).

We previously reported that the transplant subjects, who are prophylactically treated with the antibiotic sulfamethoxazole-trimethoprim (Bactrim), developed potential resistance to the antibiotic by increases in dihydrofolate synthase/folylpolyglutamate synthase, two enzymes that can increase folate production but are not blocked by sulfamethoxazole-trimethoprim [5]. Furthermore, our analysis of functions in the SEED database using MG-RAST showed increased “Resistance to antibiotics and toxic compounds” in the transplant group (see Figure 3). Based on these observations, we analyzed antibiotic resistance genes in the transplant and control metagenomes that demonstrated an increased abundance of ARGs as well as more diversity of ARGs in the transplant group (Figure 2). For example, out of 44 different ARGs detected, 29 were unique to the transplant subjects (Figure 4). Also, we detected an average of 16 different ARGs in transplant group versus only 2 in control. Interestingly, the putative specificity of the antibiotic resistance was also different. In the control subjects, resistance was predicted to bactrim, chloramphenicol, erythromycin, fluoroquinolone, fosmidomycin, penicillin, and tetracycline, all antibiotics that are previously or currently used in clinical or agricultural practice. In contrast, the transplant subjects projected resistance to aminoglycosides, cephalosporins, fosfomycin, norfloxacin, kasugamycin, lincomycin, macrolides, and polymyxin antibiotics that are often administered for more serious clinical indications. The most striking observation in our study was the type of ARGs that were detected in the transplant versus control subjects. 18 of the 36 ARGs in the transplant group were classified as multidrug efflux pumps (MDEPs), whereas only 2 were detected at a significant level in the control group. SIMPER analysis showed that MDEPs contributed 65.7% towards dissimilarity among the groups.

The MDEPs differ in the multiple aspects from the other ARGs. There are five major types of MDEPs including ABC (ATP binding cassette), MATE (toxic compound extrusion family), MFS (major facilitator super family), SMR (small multidrug resistance family), and RND (resistance/nodulation division family) [8, 9]. We identified MDEPs from 4 of the 5 types of efflux pumps including 2, 0, 8, 1, and 6 from the ABC, MATE, MFS, SMR, and RND types, respectively (Figure 7(c)). Thus, the MDEPs were derived from multiple diverse types of efflux pumps. MFS multidrug efflux pumps genes (*emea*, *emrd*, *mdtg*, *mdth*, *mdtk*, *mdtl*, *mdtm*, *tetc*), and RND (*acrb*, *macb*, *mdte*, *mdtf*, *mdto*, *tolc*), were among the abundant antibiotic classes compared to ABC (*bcr*, *isa*) and SMR (*emre*). Of note *mdtk*, *acrb*, and *bcr* were highly abundant among the MFS, RND, and ABC, respectively (Table 2, Figure 7(c)). The MDEPs are ancient elements present in prokaryotes, archaebacteria, and eukaryotes that exert important biological functions unrelated to antibiotic resistance including quorum sensing, virulence, detoxification, and biofilm formation. In cancer subjects, the MDEPs can promote resistance to chemotherapy agents. In bacteria, the MDEPs can promote resistance to antibiotics. The MDEP genes are commonly encoded within the genome and structural components are highly conserved in all members of a given bacterial species and subject to low rates of mutation. A single MDEP can convey resistance to multiple antibiotics. Since the MDEPs are ancient genes it is likely that antibiotic resistance by MDEPs is an emergent property not dependent on recent mutations suggesting that MDEPs are not selected due to exposure to antibiotics. In contrast, the development of resistance to higher concentrations or new structures in plasmid encoded ARGs is rapid and can occur within hours to days [14].

Our data support the hypothesis that bacteria with diverse abundant MDEPs have increased fitness to colonize and survive in a high stress environment in a transplant host. Transplantation produces multiple stressors including ischemia/reperfusion, exposure to antibiotics, and immunosuppressive agents. Thus, microbiota with relevant MDEPs may have increased fitness and provide the bacteria with a selective advantage in a stressed environment such as induced by transplantation. This could guide the specific changes in the microbiome we documented following transplantation. Further delineation of the effects of high levels of MDEPS in transplant subjects' merits further investigation.

## Figures and Tables

**Table 1:** Relative abundance of bacterial genus in Transplant and Control group. Genera are selected on the basis of >1% abundance in each group. Genera identified in both groups are shown in bold font. The significance of the difference among bacterial genera was computed using Students *t*-test (^*∗*^*p* ≤ 0.05).

Transplant		Control	
Genus	% Abundance	Genus	% Abundance
**Enterococcus ** ^*∗*^	**28.6**	**Ralstonia ** ^*∗*^	**33.2**
**Escherichia ** ^*∗∗*^	**12.7**	**Propionibacterium ** ^*∗*^	**10.9**
**Ralstonia ** ^*∗*^	**9.6**	**Enterococcus ** ^*∗*^	**7.2**
**Shigella ** ^*∗∗*^	**7.4**	**Neisseria ** ^*∗*^	**7.1**
Propionibacterium^*∗*^	4.8	Bifidobacterium	5.0
Proteus	4.4	Corynebacterium^*∗*^	3.8
Streptococcus	3.4	Streptococcus	2.5
Lactobacillus	2.9	Lactobacillus	2.3
Bacteroides	2.4	Burkholderia	2.0
**Neisseria ** ^*∗*^	**2.2**	Cupriavidus	1.7
Salmonella^*∗*^	1.6	Bacteroides	1.7
Burkholderia	1.3	**Prevotella ** ^*∗∗*^	**1.4**
**Staphylococcus ** ^*∗*^	**1.2**	Pseudomonas	1.1
Citrobacter	1.1	**Mobiluncus ** ^*∗*^	**1.1**

**Table 2:** SIMPER (Similarity percentage analysis) for the transplant and control group. Contribution % towards dissimilarity in both groups is shown above 1%.

Antibiotic class	Gene	Target	Transplant (mean abundance)	Control (mean abundance)	Contribution (%)
Erythromycin	lsa	ABC	0.18	0.01	5.5
Bacitracin	bcr	ABC	0.54	0.00	3.9
Tetracycline	tetc	EF	0.22	0.00	2.1
Macrolide	macb	MEP	0.46	0.00	2.9
Fluoroquinolone	emea	MEP	0.19	0.00	7.5
Multidrug efflux pump	acrb	MEP	0.64	0.00	7.3
Deoxycholate, fosfomycin	mdtf	MEP	0.31	0.00	4.6
Enoxacin, norfloxacin	mdtk	MEP	0.65	0.00	4.2
Chloramphenicol	mdtl	MEP	0.39	0.00	3.4
Chloramphenicol	ceob	MEP	0.00	0.01	3.1
Multidrug efflux pump	mdfa	MEP	0.40	0.00	3.1
Deoxycholate, fosfomycin	mdth	MEP	0.22	0.00	2.9
Deoxycholate, fosfomycin	mdtg	MEP	0.42	0.00	2.8
Multidrug efflux pump	mdto	MEP	0.36	0.00	2.8
Multidrug efflux pump	mexb	MEP	0.00	0.00	2.8
Multidrug efflux pump	tolc	MEP	0.19	0.00	1.5
Multidrug efflux pump	emre	MEP	0.19	0.00	1.4
Multidrug efflux pump	amrb	MEP	0.00	0.00	1.2
Multidrug efflux pump	mdtm	MEP	0.14	0.00	1.2
Multidrug efflux pump	aph3iiia	Phosphorylation	0.05	0.00	1.5
*Sum*			*5*.*55*	*0*.*02*	*65*.*7*
Erythromycin	ermb	23S rRNA	0.08	0.00	4.1
Erythromycin	ermx	23S rRNA	0.00	0.03	1.4
Kasugamycin	ksga	30S	0.62	0.00	5.2
Polymyxin	arna	Arabinose	0.68	0.00	4.9
Cephalosporin, penicillin	bl1_ec	*β*-Lactam	0.64	0.00	4.7
Cephalosporin, penicillin	bl2b_tem	*β*-Lactam	0.41	0.00	2.2
Penicillin	pbp2b	Glycosylase	0.00	0.02	1.2
Bacitracin	baca	Phosphatase	0.53	0.02	7.9
*Sum*			*2*.*96*	*0*.*07*	*31*.*6*

## Data Availability

The sequence data for the study have been submitted to the MG-RAST web server under the following accession number provided in [Supplementary-material supplementary-material-1].

## Supplementary Materials

Supplementary Materials Figure S1: ANOSIM statistics distance plots. (a) Transplant male vs. female, (b) control male vs. female, (c) transplant group vs. control group. Table S1: demographics of transplant and control subjects. Transplant samples (T1–T21) and control samples (C1–C8) have been previously analyzed [5]. Table S2: sequencing statistics and accession numbers. Table S3: taxonomic and functional annotation in transplant and control groups.
