# Supplementary material for: Abundance of Multidrug Resistance Efflux Pumps in the Urinary Metagenome of Kidney Transplant Patients
Source: Biomed Res Int. 2020 Mar 12;2020:5421269. doi: 10.1155/2020/5421269 (PMC7093903; doi:10.1155/2020/5421269)
Supplement: Supplementary Materials — Figure S1: ANOSIM statistics distance plots. (a) Transplant male vs. female, (b) control male vs. female, (c) transplant group vs. control group. Table S1: demographics of transplant and control subjects. Transplant samples (T1–T21) and control samples (C1–C8) have been previously analyzed [5]. Table S2: sequencing statistics and accession numbers. Table S3: taxonomic and functional annotation in transplant and control groups. [file 5421269.f1.zip › 5421269.f1/ARG_0804_Supplementary-Data_0217.pdf]

SUPPLEMENTARY DATA: Supplementary Figures and Tables

Abundance of Multidrug Resistance Efflux Pumps in the Urinary Metagenome of Kidney Transplant Patients

Asha Rani[1*^], Ravi Ranjan[1*@], Ahmed A. Metwally[1,2], Daniel C. Brennan[3], Patricia W. Finn[2,4,5#], David L. Perkins[2,6,7#]

[1]Department of Medicine, University of Illinois at Chicago, IL 60612 USA
[2]Department of Bioengineering, University of Illinois at Chicago, IL 60612 USA
[3]Department of Medicine, Johns Hopkins School of Medicine, Baltimore, MD 21287 USA
[4]Division of Pulmonary, Critical Care, Sleep, and Allergy, Department of Medicine, University of Illinois at Chicago, IL 60612 USA
[5]Department of Microbiology and Immunology, University of Illinois at Chicago, IL 60612 USA
[6]Division of Nephrology, Department of Medicine, University of Illinois at Chicago, IL 60612 USA
[7]Department of Surgery, University of Illinois at Chicago, IL 60612 USA

*Corresponding author:
David Perkins, MD, PhD
University of Illinois at Chicago
Department of Medicine, MC 787
840 S Wood Street, Suite 1020N CSB
Chicago IL 60612 USA
Email: perkinsd@uic.edu
Phone: 312-413-3382, Fax: 312-355-0499

* Equal first author contribution, # Equal last author contribution.

^Current Address: Asha Rani, Department of Food Science, University of Massachusetts, Amherst MA 01003 USA

@Current Address: Ravi Ranjan, Genomics Resource Laboratory, Institute for Applied Life Sciences (IALS), University of Massachusetts, Amherst MA 01003 USA

Running title:
Human urinary resistome

Supplementary Figures and Table Legends

Figure S1. ANOSIM statistics distance plots. (a) Transplant male *vs* female, (b) Control male *vs* female, (c) Transplant group *vs* Control group.

Table S1. Demographics of transplant and control subjects. Transplant samples (T1-T21) and Control samples (C1-C8) have been previously analyzed (Rani et al 2017).

Table S2. Sequencing statistics and accession numbers.

Table S3. Taxonomic and functional annotation in transplant and control groups.

Figure S1. ANOSIM statistics distance plots. (a) Transplant male *vs* female, (b) Control male *vs* female, (c) Transplant group *vs* Control group.

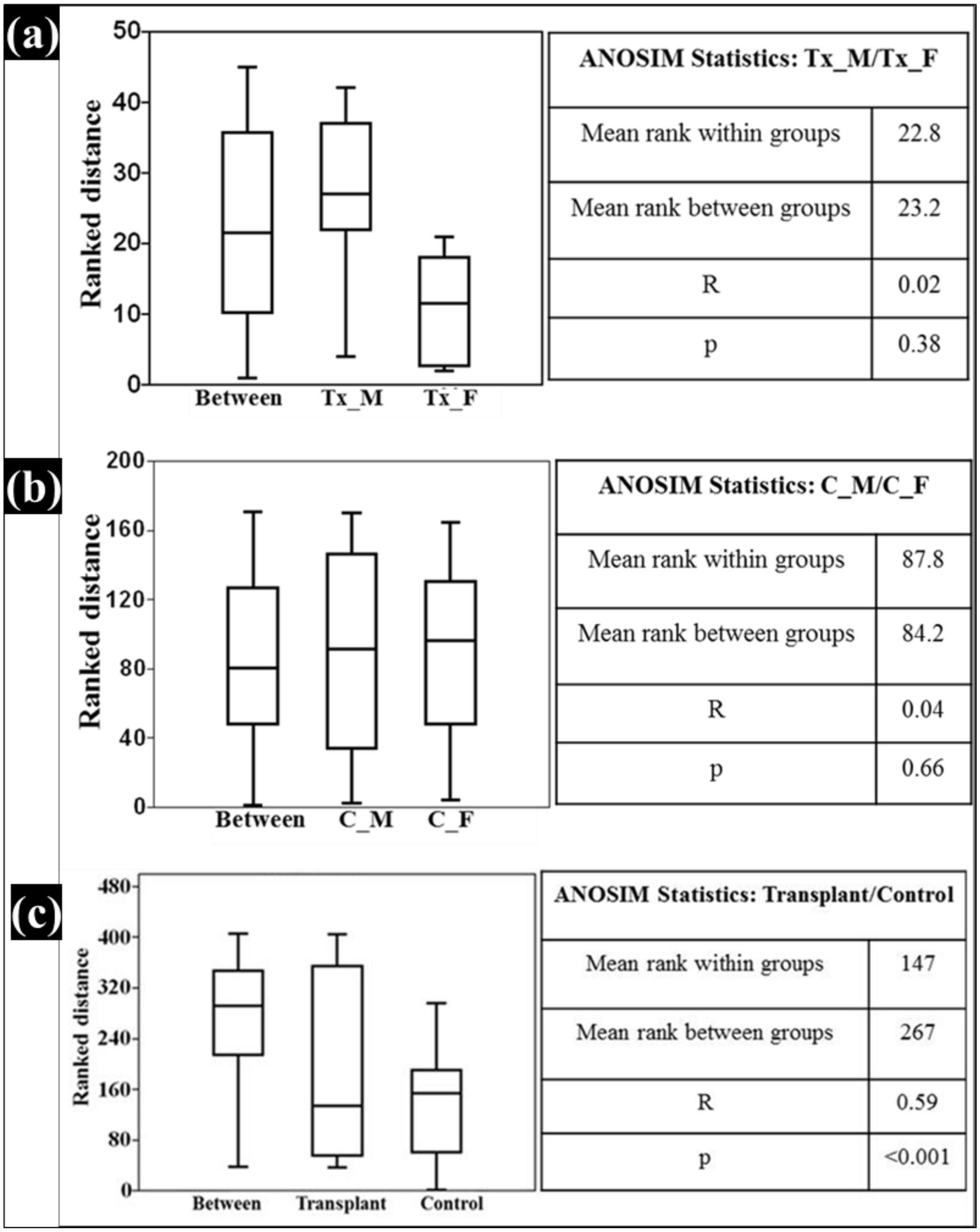

Table S1. Demographics of transplant and control subjects. Transplant samples (T1-T21) and Control samples (C1-C8) have been previously analyzed (Rani et al 2017).

Sample number	Age (Years)	Gender	Ethnicity	Clinical Diagnosis	Calcineurin Inhibitor
T1	59	M	Caucasian	T1DM	FK
T2	76	M	African American	T2DM	FK
T3	31	M	Caucasian	PSGN	FK
T4	47	M	Caucasian	PKD	CYA
T5	59	M	Caucasian	HTN	FK
T6	65	M	Caucasian	HTN	FK
T7	66	M	Caucasian	T2DM	FK
T8	52	M	African American	HTN	CYA
T9	62	F	African American	T1DM	FK
T10	40	F	Caucasian	Vasculitis	CYA
T11	45	F	African American	FSGS	CYA
T12	51	M	Caucasian	T2DM	FK
T13	41	M	Caucasian	HTN	FK
T14	51	F	Caucasian	Lupus	FK
T15	46	F	Caucasian	RN	FK
T16	54	M	Caucasian	HTN	FK
T17	74	F	Caucasian	HTN	FK
T18	46	M	Caucasian	HTN	FK
T19	54	M	Caucasian	PKD	FK
T20	32	F	Caucasian	T1DM	FK
T21	68	M	Caucasian	Nephrocalcinosis	FK
C1	31	F	Asian	-	-
C2	31	F	Caucasian	-	-
C3	30	F	Caucasian	-	-
C4	29	M	Asian	-	-
C5	32	F	Asian	-	-
C6	30	M	Caucasian	-	-
C7	63	M	Caucasian	-	-
C8	27	F	Caucasian	-	-
C9	38	M	Caucasian	-	-
C10	24	M	African American	-	-
C11	34	M	Caucasian	-	-
C12	39	F	Caucasian	-	-
C13	37	F	Caucasian	-	-
C14	30	M	Caucasian	-	-
C15	30	M	Caucasian	-	-
C16	23	F	Caucasian	-	-
C17	45	M	African American	-	-
C18	40	M	Caucasian	-	-
C19	36	F	Caucasian	-	-
C20	31	M	Caucasian	-	-
C21	25	M	Caucasian	-	-
C22	40	M	Caucasian	-	-
C23	35	M	Caucasian	-	-
C24	40	F	African American	-	-
C25	35	F	Caucasian	-	-

Table S2. Sequencing statistics and accession numbers.

Sample name	No. of reads	No. of contigs	MG-RAST ID
T1	7,849,916	47	4504962.3
T2	21,803,762	2,490	4532153.3
T3	10,327,248	25	4505561.3
T4	12,785,361	14	4505562.3
T5	7,433,295	374	4505340.3
T6	8,174,663	312	4505341.3
T7	34,818,382	7,487	4524189.3
T8	27,420,614	4,935	4524191.3
T9	31,903,768	2,417	4524193.3
T10	28,570,804	6,376	4524195.3
T11	35,156,390	5,503	4524197.3
T12	642,591	142,377	4554299.3
T13	392,172	17,351	4559944.3
T14	281,816	116,106	4554300.3
T15	399,222	103,700	4554302.3
T16	580,114	120,382	4554303.3
T17	689,472	179,096	4554304.3
T18	881,827	224,869	4554305.3
T19	446,857	131,806	4554306.3
T20	563,269	78,559	4554307.3
T21	295,740	72,108	4554308.3
Total	231,417,283	1,216,334	

Sample name	MG-RAST ID	No. of reads	No. of contigs
C1	4555679.3	7,545,864	10,732
C2	4555680.3	5,384,830	14,516
C3	4556080.3	9,235,514	15,563
C4	4555681.3	9,790,696	16,247
C5	4555682.3	8,187,139	13,343
C6	4556081.3	8,043,242	5,387
C7	4557780.3	4,690,991	6,946
C8	4557851.3	3,756,888	7,507
C9	4641627.3	1,510,882	8,346
C10	4641628.3	596,298	3,542
C11	4641629.3	1,830,274	9,312
C12	4641630.3	1,463,490	3,409
C13	4641631.3	1,838,588	9,073
C14	4641632.3	1,748,344	11,604
C15	4641633.3	602,874	3,566
C16	4641634.3	1,757,026	9,525
C17	4641635.3	789,210	4,992
C18	4641636.3	2,843,574	16,150
C19	4641637.3	1,296,320	5,407
C20	4641638.3	1,979,906	11,937
C21	4641639.3	2,078,520	6,089
C22	4641640.3	1,167,598	4,270
C23	4641641.3	807,426	3,053
C24	4641642.3	635,262	3,540
C25	4641643.3	1,621,126	8,943
Total		81,201,882	212,999
